# Supplementary material for: Drug repurposing for COVID-19 using graph neural network and harmonizing multiple evidence
Source: Sci Rep. 2021 Nov 30;11:23179. doi: 10.1038/s41598-021-02353-5 (PMC8632883; doi:10.1038/s41598-021-02353-5)

Supplementary Figure S1. The SARS-CoV-2 Knowledge Graph

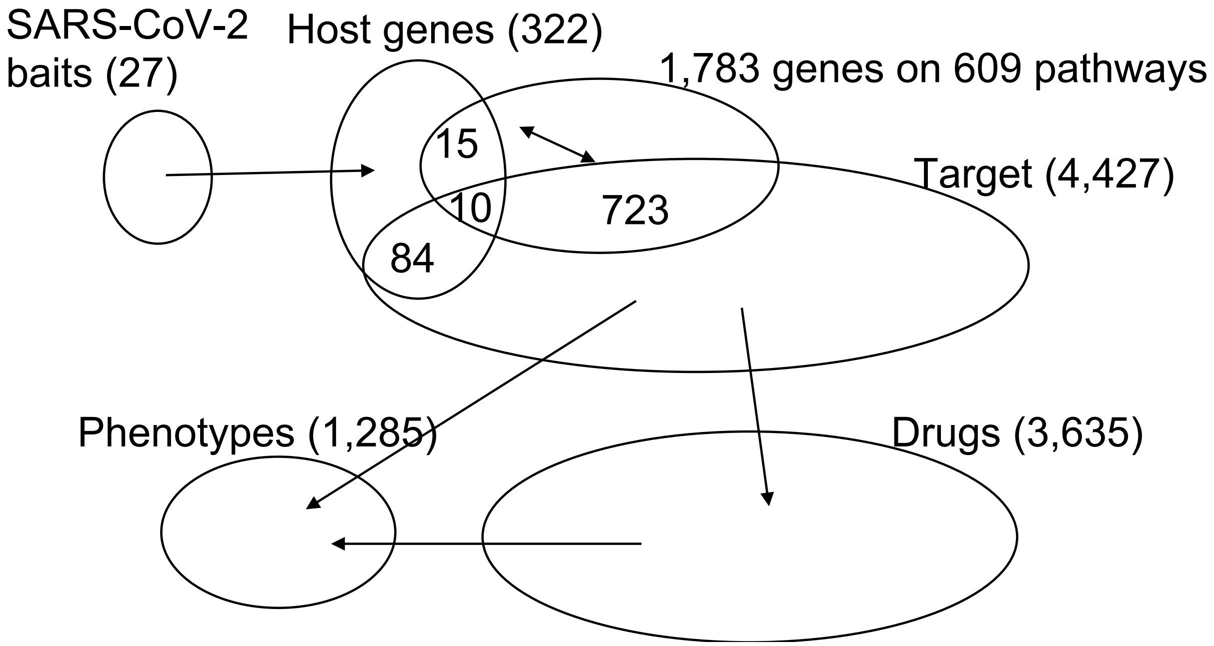

Supplementary Figure S2. Interactive t-sne plot (Created by Plotely (https://plotly.com/))

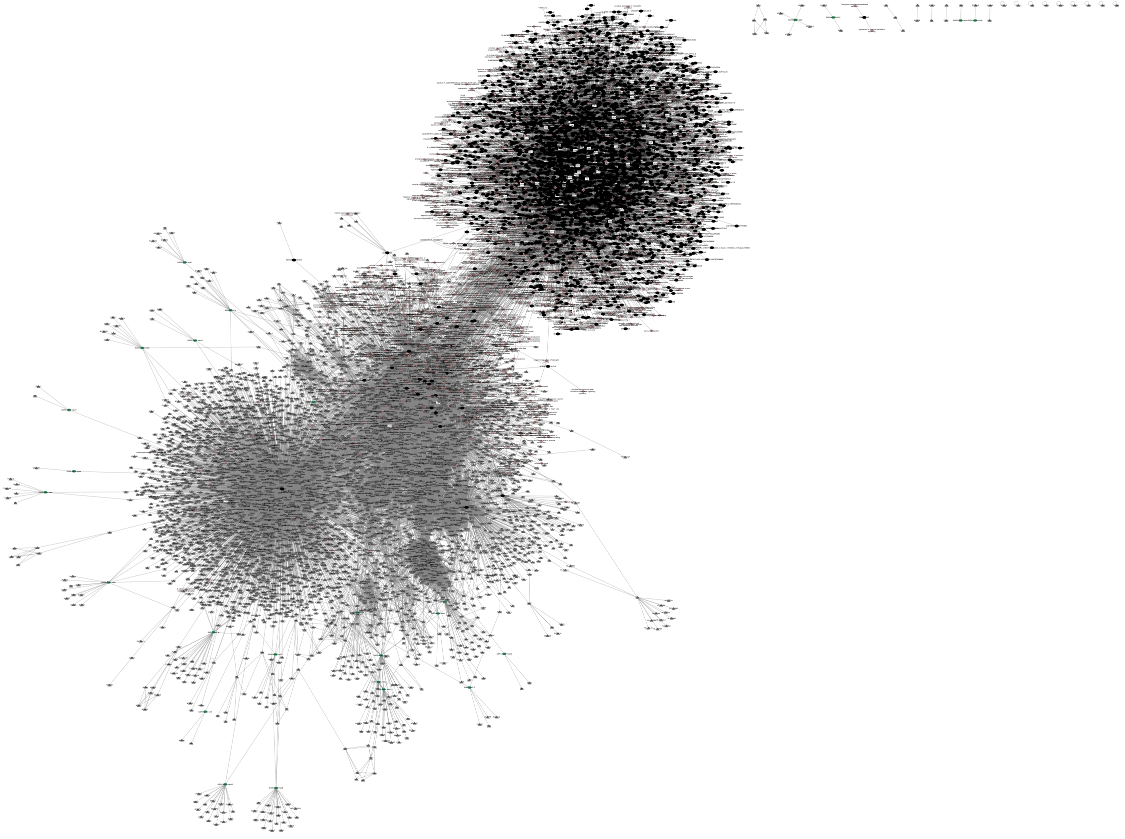

Supplementary Figure S3. External validation (a) Accuracy was measured in the intersection of candidate drugs and external validation sources. (b) Cohort selection and propensity score matching in EHRs.

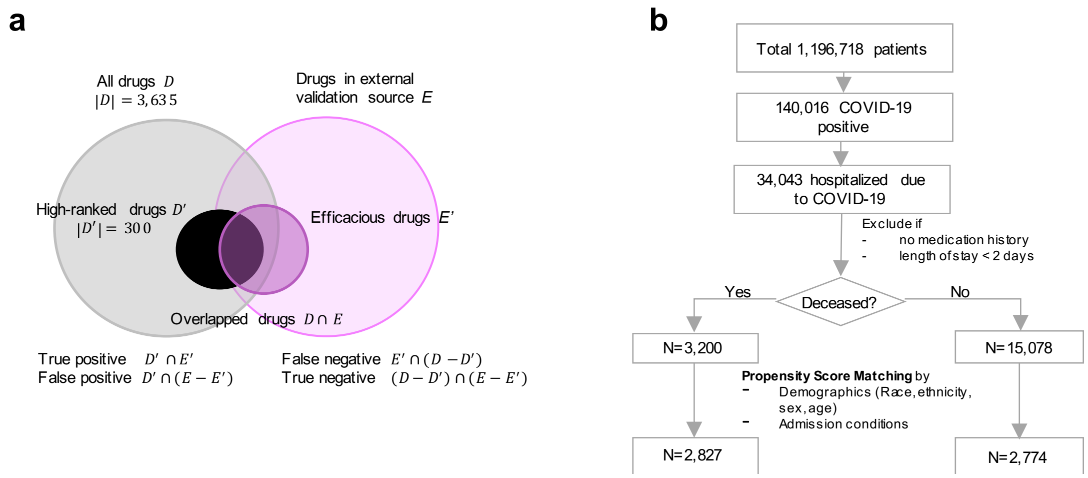

Supplement: Supplementary file 1 — Supplementary Figures. [file 41598_2021_2353_MOESM1_ESM.pdf]
