# Supplementary material for: Drug repurposing for COVID-19 using graph neural network and harmonizing multiple evidence
Source: Sci Rep. 2021 Nov 30;11:23179. doi: 10.1038/s41598-021-02353-5 (PMC8632883; doi:10.1038/s41598-021-02353-5)
Supplement: Supplementary file 3 — Supplementary Tables. [file 41598_2021_2353_MOESM3_ESM.pdf]

Table S1. Link prediction accuracy in the SARS-CoV-2 knowledge graph.

	AUROC	AUPRC
SARS-CoV-2 knowledge graph embedding	0.8132	0.8536
General embedding (*17*)	0.5695	0.6431
SARS-CoV-2 knowledge graph embedding + general embedding (proposed)	0.8121	0.8524

Table S2. COVID-19 hospitalized patient's demographics and comorbidities before and after PSM.

	Before matching		After matching	
	Recovered	Deceased	Recovered	Deceased
Number of patients	15,078	3,200	2,774	2,827
Age				
Mean	60.10	73.78	73.64	73.24
Standard deviation	17.63	12.81	12.95	12.86
Sex				
Male	7,765	1,887	1,601	1,630
Female	7,309	1,313	1,172	1,197
Race				
Caucasians	7336	2031	1728	1790
African Americans	4052	544	511	490
Asian Americans	470	113	97	102
Others	3,220	512	438	445
Admission conditions				
Temperature	36.93	37.16	37.07	37.00
SPO_2	94.21	91.39	92.32	92.56

Supplementary Table S3. ATT score of 138 drugs that were in EHRs and initial 3,635 drugs.

Drug Name	pvalue (ATT)	t-stats (ATT)	treatment coefficient (ATT)	pvalue (unweighted)	t-stats (unweighted)	treatment coefficient (unweighted)	Total patients receiving the drugs	Decease	Recovered
ACETAMINOPHEN	1.52E-24	10.27389744	0.252072626	0.550702813	-0.596743439	-0.014009005	5103	2582	2521
CEFTRIAXONE	2.28E-38	13.05060256	0.210259862	0.407964106	0.827543924	0.01107434	2951	1474	1477
AZITHROMYCIN	1.47E-38	13.08483476	0.181604712	1.95E-08	-5.624915178	-0.075312701	3072	1655	1417
HEPARIN	0.49937998	0.675509555	0.025875993	2.75E-58	-16.28408353	-0.21272357	2721	1671	1050
ATORVASTATIN	0.006503854	2.722256456	0.17	0.383479597	0.871571421	0.012083802	2054	1021	1033
ALBUTEROL	2.94E-08	5.552787653	0.142186258	0.000298277	-3.619067212	-0.048326012	2723	1442	1281
ASPIRIN	0.020407976	2.319421802	0.135778789	0.694861662	-0.392286238	-0.005335267	2280	1158	1122
LISINOPRIL	0.01856643	2.354811561	0.086919475	3.81E-11	6.624366208	0.149011795	543	201	342
METOPROLOL	0.336169089	-0.961844834	-0.030411324	1.62E-28	-11.13858611	-0.162064538	1630	1010	620
HYDROXYCHLOROQUINE	0.001458711	3.184357039	0.076521131	2.59E-06	-4.705464527	-0.063183845	2476	1337	1139
GLUCAGON	9.86E-06	4.424259631	0.076512073	1.95E-15	-7.967147395	-0.107978514	2250	1281	969
METHYLPREDNISOLONE	0.32935996	-0.975488876	-0.057664382	2.37E-17	-8.50149031	-0.136986469	1214	743	471
AMLODIPINE	0.080016741	1.750906708	0.131551424	1.51E-06	4.814998349	0.078394582	1194	529	665
NALOXONE	0.02934147	2.179427378	0.070713533	0.002352089	-3.043140751	-0.052405111	1032	565	467
MELATONIN	0.334340181	0.965491918	0.034430552	0.021603329	2.297914841	0.038525834	1108	525	583
VANCOMYCIN	0.007165021	-2.690064133	-0.079295125	3.12E-148	-26.72867558	-0.347059814	2111	1522	589
FUROSEMIDE	0.016089307	-2.407613634	-0.087255464	2.00E-86	-20.05121062	-0.259884248	2548	1647	901
CLOPIDOGREL	0.016115311	-2.407023299	-0.102858935	0.076550381	-1.771389254	-0.041957097	488	265	223
ASCORBIC ACID	0.48643081	-0.696042971	-0.032217096	0.029570266	-2.17635658	-0.039249477	919	494	425

DOXYCYCLINE	0.810558636	0.239716735	0.006825173	0.087643702	-1.708259119	-0.03128549	885	470	415
LIDOCAINE	0.555760541	-0.589185608	-0.019581957	3.35E-05	-4.151845808	-0.076833462	860	490	370
HYDRALAZINE	0.88880536	-0.13982238	-0.007041279	3.33E-05	-4.15312725	-0.078065173	828	473	355
FAMOTIDINE	0.007344525	-2.681785537	-0.107907601	2.21E-82	-19.5494306	-0.278437652	1626	1142	484
LIDOCAINE	0.076929572	-1.769112262	-0.062406859	1.43E-15	-8.006367659	-0.152743836	791	503	288
AMIODARONE	0.02153417	-2.299130532	-0.108665461	4.15E-43	-13.88264747	-0.288463905	631	480	151
METOPROLOL	0.171384486	1.367944053	0.049362437	9.48E-05	3.906286791	0.080739401	662	287	375
CHOLECALCIFEROL	0.105939157	-1.61697896	-0.054344712	0.194198821	-1.298413322	-0.027248251	641	339	302
IOHEXOL	0.725380857	-0.35129421	-0.014653047	0.972671424	0.034259522	0.000733244	613	309	304
PREDNISONE	0.488183782	0.693246252	0.077984554	0.000209746	3.709420455	0.082393629	562	242	320
CEFEPIME	0.005685268	-2.766470176	-0.113959176	8.56E-61	-16.65028399	-0.271604739	1119	808	311
DEXAMETHASONE	0.033852598	-2.122344484	-0.124416688	9.37E-07	-4.90987005	-0.138242975	334	212	122
ATROPINE	0.022120691	-2.288926452	-0.130315553	4.11E-24	-10.17605311	-0.259251708	408	304	104
DIPHENHYDRAMINE	0.647715175	-0.456963269	-0.029131586	0.163931215	-1.392153487	-0.032978057	488	261	227
NITROGLYCERIN	0.043741949	-2.017011523	-0.141978747	0.398616665	-0.844158934	-0.023952674	330	174	156
FINASTERIDE	0.011680759	-2.522473603	-0.144157031	0.821870312	0.225150629	0.006823079	287	143	144
LABETALOL	0.86450268	-0.170653137	-0.011344517	1.36E-07	-5.276866415	-0.129787772	447	279	168
LOSARTAN	0.812240078	0.237548387	0.010650863	6.23E-08	5.419494879	0.133826172	443	169	274
HYDROCORTISONE	0.000104209	-3.883361007	-0.153012582	1.11E-47	-14.64408789	-0.36621866	416	351	65
CARVEDILOL	0.245984493	-1.160279516	-0.055117877	0.920029449	-0.100401154	-0.002536163	424	215	209
HYDROCHLOROTHIAZIDE	0.037351158	-2.082400873	-0.161644983	0.000661339	3.407040484	0.120825648	206	80	126
OLANZAPINE	0.10617452	-1.61588962	-0.092136952	8.93E-05	-3.920908882	-0.104419106	377	227	150
METOCLOPRAMIDE	0.854962385	-0.182798958	-0.013293492	0.000203579	-3.716989264	-0.099246843	375	224	151
LACTULOSE	0.362853861	-0.910014831	-0.058763804	2.06E-10	-6.368402754	-0.171346124	367	244	123
DEXMEDETOMIDINE	7.05E-05	-3.977690657	-0.169460654	1.29E-81	-19.45298005	-0.336336219	942	739	203
IOPAMIDOL	0.010134272	-2.572087975	-0.173839037	0.729095019	0.346346928	0.010461396	289	143	146
SERTRALINE	0.00581868	-2.758888076	-0.177697332	0.586720715	-0.5436257	-0.016132805	300	156	144

HALOPERIDOL	3.75E-07	-5.087154213	-0.185116261	1.13E-31	-11.78253894	-0.243797278	644	464	180
PRAVASTATIN	0.036424225	-2.092662207	-0.187475611	0.008566573	2.629812292	0.093520042	205	85	120
FOLIC ACID	0.000384143	-3.552903448	-0.190015468	0.051613944	-1.946755946	-0.054456768	340	189	151
IBUPROFEN	0.008138237	-2.647226135	-0.199536208	0.006474995	2.723727615	0.100722674	189	77	112
LIDOCAINE	0.516998696	0.648019831	0.061570746	0.002099478	3.077224189	0.087371028	329	139	190
KETAMINE	0.008692101	-2.624856637	-0.20157909	1.15E-50	-15.12195802	-0.344262655	510	417	93
TRAMADOL	0.638517053	0.469798821	0.022322133	0.00780635	2.661292504	0.076907371	317	137	180
METRONIDAZOLE	0.283333	-1.072965139	-0.062391292	1.82E-06	-4.777592291	-0.140182192	306	195	111
LOPERAMIDE	0.753752489	-0.313710662	-0.030289362	0.001760601	3.129406792	0.092500272	302	126	176
SIMVASTATIN	0.3622305	-0.911197591	-0.055411681	0.003743923	2.90018638	0.086004527	300	127	173
MONTELUKAST	0.01230472	-2.504095197	-0.210752335	0.82778542	0.217552892	0.007052875	249	124	125
DONEPEZIL	0.007004354	-2.697633505	-0.212247981	0.664626221	-0.433557892	-0.014109546	247	128	119
PHENYLEPHRINE	2.15E-08	-5.607968211	-0.219606743	8.92E-81	-19.34698748	-0.342976524	886	703	183
FENTANYL	0.008933633	-2.615498585	-0.220146132	1.31E-21	-9.58830844	-0.344965376	197	165	32
NYSTATIN	0.327811817	-0.978616434	-0.051090749	0.160598378	-1.403247912	-0.043842745	269	147	122
ALPRAZOLAM	0.00041075	-3.535206744	-0.222022157	0.728290678	0.347417592	0.011350231	245	121	124
WARFARIN	0.411922267	-0.820576859	-0.046739625	0.354669986	-0.925645783	-0.029449008	259	138	121
DIGOXIN	0.071037094	-1.805583614	-0.126615001	2.65E-11	-6.678373715	-0.213617467	254	180	74
REMDESIVIR	0.154111937	-1.425349512	-0.138925953	0.285393747	-1.06838321	-0.034370649	253	136	117
DOBUTAMINE	0.012132084	-2.509095841	-0.23446039	2.47E-14	-7.643409088	-0.395523686	94	84	10
BENZOCAINE	0.558394023	-0.585263673	-0.065314054	0.015094551	2.43085205	0.079068357	247	106	141
CETIRIZINE	0.043594867	-2.018422509	-0.23865077	0.610148318	-0.509890439	-0.025728049	100	53	47
ACETYLCYSTEINE	0.432469607	-0.785029296	-0.051994277	1.97E-15	-7.966229766	-0.273069033	219	168	51
EPINEPHRINE	0.007593828	-2.670583981	-0.240864967	5.64E-65	-17.24542686	-0.399637329	484	421	63
FENTANYL	8.13E-13	-7.175685094	-0.249212424	4.82E-146	-26.51571916	-0.367715815	1629	1247	382
ACETAZOLAMIDE	1.86E-06	-4.773348846	-0.261703315	4.71E-05	-4.072860966	-0.152450819	184	120	64
OMEPRAZOLE	0.075037833	-1.780564494	-0.12581213	4.67E-05	4.074976322	0.145471685	203	74	129
BUDESONIDE	0.001976583	-3.095189389	-0.266768465	0.00170561	-3.138734458	-0.141304631	126	81	45
CLONAZEPAM	0.738489419	0.333870985	0.034185014	0.028981691	-2.184298105	-0.084087862	174	102	72

ARIPIPRAZOLE	0.034036056	-2.120164267	-0.267117454	0.682198974	-0.409485631	-0.024983787	68	36	32
DIAZEPAM	0.462866085	-0.734186072	-0.058823735	0.001304878	-3.216532754	-0.124810184	171	107	64
CLONIDINE	0.393912595	0.85260908	0.073372247	0.492551422	-0.6863015	-0.027695542	158	84	74
ADENOSINE	0.006283533	-2.733640102	-0.276743382	2.91E-12	-6.997319936	-0.351545174	100	85	15
MORPHINE	3.17E-16	-8.191278765	-0.276854178	8.67E-118	-23.63341704	-0.344477159	1441	1096	345
RIVAROXABAN	0.00296652	-2.972490377	-0.27693123	0.067272637	1.830203575	0.070860838	172	75	97
HEPARIN	0.546544727	-0.602982541	-0.092703765	1.22E-06	-4.858475454	-0.203998129	145	102	43
LEVOFLOXACIN	1.11E-05	-4.39839493	-0.291032643	0.000186503	-3.739127938	-0.115484245	275	169	106
PROPOFOL	2.91E-16	-8.20201345	-0.293547662	3.34E-181	-29.79514826	-0.406610968	1638	1298	340
NOREPINEPHRINE	2.89E-06	-4.683234443	-0.324725396	7.66E-219	-33.03791569	-0.45085335	1556	1292	264
NIFEDIPINE	0.83811023	0.204320817	0.047122956	0.004360357	2.852004747	0.125998856	131	50	81
DEXTROMETHORPHAN	0.984564408	-0.019347717	-0.003307988	0.706113854	0.377099636	0.016926113	127	62	65
METFORMIN	0.173248537	1.362013122	0.128268375	1.31E-07	5.284949271	0.243276774	120	32	88
TORSEMIDE	0.213017286	-1.245453307	-0.104647901	0.702274631	0.382271325	0.017712667	119	58	61
SCOPOLAMINE	2.15E-06	-4.743444854	-0.333392244	6.27E-57	-16.08258777	-0.435813416	347	317	30
ATENOLOL	0.001131852	-3.257206171	-0.35030926	0.170725927	1.370050963	0.057254365	147	66	81
MORPHINE	0.030879046	-2.15917354	-0.353729512	9.00E-05	-3.918934659	-0.182848941	117	80	37
VANCOMYCIN	0.009644346	-2.589216837	-0.356777498	0.000130297	-3.828568688	-0.187496352	106	73	33
MEMANTINE	0.001216154	-3.236721041	-0.359497246	0.425299365	-0.797319403	-0.030705349	174	93	81
FENOFIBRATE	0.041089964	-2.043092912	-0.36964403	0.935187321	-0.081323841	-0.005956987	47	24	23
NICARDIPINE	0.528635809	-0.630129142	-0.069673345	1.20E-06	-4.861234975	-0.240111739	104	77	27
ROPINIROLE	0.029027219	-2.183678866	-0.445238313	0.116196113	-1.571185324	-0.10741542	54	33	21
SUCRALFATE	0.647125729	-0.457783537	-0.075371012	0.044144648	-2.013168774	-0.101051305	101	61	40
SPIRONOLACTONE	2.00E-05	-4.268228553	-0.46120459	0.343718672	-0.946923411	-0.041706145	132	72	60
HYDROXYCHLOROQUINE	0.009454214	-2.596073867	-0.463961709	0.079140062	-1.756017669	-0.085690762	107	63	44
GLUCAGON	0.018680633	-2.352529535	-0.480747195	0.553534941	-0.592507181	-0.04822748	38	21	17
HYDROCORTISONE	0.84177018	0.199638993	0.023694268	0.406131154	0.830783881	0.043906185	91	42	49
PROMETHAZINE	0.000111859	-3.866063104	-0.580121488	0.69716127	-0.389175305	-0.018996084	107	56	51

NICOTINE	0.09910498	-1.649482217	-0.223853891	0.598164365	0.527072154	0.028975375	84	40	44
FLUOXETINE	1.06E-06	-4.885335236	-0.612301139	0.04563578	-1.999192464	-0.095390456	112	67	45
VALSARTAN	0.15097006	-1.436309858	-0.177813472	0.192515282	1.303331266	0.072064263	83	36	47
HYDROXYZINE	0.639695012	-0.468150749	-0.090602994	0.446780142	0.760847597	0.042843235	80	37	43
CIPROFLOXACIN	0.856658274	0.180637988	0.040885185	0.668801062	0.427816658	0.02454931	77	37	40
EZETIMIBE	7.27E-05	-3.970189916	-0.632547161	0.346960471	0.94058047	0.04997883	90	41	49
ARGATROBAN	0.323169107	-0.988053573	-0.143882152	1.86E-14	-7.680716018	-0.447121991	74	70	4
BACLOFEN	3.15E-05	-4.166052009	-0.632837853	0.318811224	-0.996992524	-0.049578487	103	57	46
CEFTRIAXONE	0.000408681	-3.536544221	-0.667503936	0.279253394	1.082102933	0.059834845	83	37	46
CITRIC ACID	0.21153297	-1.249504139	-0.144758184	4.40E-07	-5.056774499	-0.307829494	68	55	13
VANCOMYCIN	2.04E-08	-5.61694191	-0.673815497	8.61E-09	-5.764909199	-0.247749684	138	103	35
NITROGLYCERIN	0.703815765	-0.380194093	-0.092695599	0.037668509	-2.07893755	-0.131670364	63	40	23
PREGABALIN	2.75E-05	-4.196546967	-0.696528539	0.45363936	-0.749413735	-0.039394035	92	50	42
METHYLPREDNISOLONE	0.944841059	-0.069189742	-0.026667629	0.229352707	-1.202159164	-0.076763594	62	36	26
OSELTAMIVIR	3.40E-05	-4.148281038	-0.976161047	0.01397394	-2.458705611	-0.163600289	57	38	19
DEXAMETHASONE	0.577656482	-0.556843697	-0.124203912	0.04517158	-2.003501569	-0.129994586	60	38	22
TACROLIMUS	2.29E-05	-4.237779437	-1.033267589	0.489426943	-0.691266175	-0.044144454	62	34	28
CALCITRIOL	9.04E-07	-4.917012894	-1.05147413	0.05294531	-1.935776905	-0.121658841	64	40	24
BARIUM	0.228938637	1.203228875	0.178402695	0.124501242	1.536381925	0.102263234	57	23	34
DOPAMINE	0.406086792	-0.830862405	-0.08403194	2.35E-05	-4.232740783	-0.283820688	56	44	12
PHENOBARBITAL	2.85E-12	-7.000717272	-1.073568756	1.75E-09	-6.029752303	-0.358974098	71	61	10
CLONIDINE	0.885880155	-0.143525732	-0.026576894	0.305863473	-1.024033964	-0.070017827	54	31	23
COLCHICINE	0.689561558	0.399470819	0.086901093	0.06241026	1.863741933	0.128589599	53	20	33
LANSOPRAZOLE	0.276277312	-1.088826396	-0.183703295	5.98E-05	-4.016779485	-0.282151563	51	40	11
WARFARIN	0.458203795	-0.741859151	-0.296903666	0.7232922	-0.354080085	-0.02490726	51	27	24
PAROXETINE	0.397607051	-0.845967457	-0.121571072	0.833684119	0.209988717	0.015067194	49	24	25
PROPRANOLOL	0.063235563	-1.857900768	-0.382605495	0.947513341	0.065832739	0.004772195	48	24	24

VALPROIC ACID	0.836806383	-0.20598978	-0.039278289	0.049578024	-1.96401266	-0.142321718	48	31	17
HEPARIN	9.87E-08	-5.336121975	-1.181290608	0.031401079	-2.152493899	-0.140819872	59	38	21
PHENOL	0.321082574	-0.992323605	-0.219781922	0.262786815	-1.119941302	-0.082906117	46	27	19
VERAPAMIL	0.988169394	0.014828672	0.005830468	0.608182805	0.512698013	0.03837293	45	21	24
AMITRIPTYLINE	0.946661743	0.06690246	0.027708266	0.565581852	-0.574604561	-0.045594318	40	22	18
FENTANYL	0.000280051	-3.635391368	-1.346962062	2.69E-06	-4.697666449	-0.301834645	61	49	12
ALBUTEROL	0.000153781	-3.787473647	-1.63664526	0.16317566	1.394653571	0.116591794	36	14	22
TRIAMCINOLONE ACETONIDE	0.869917478	-0.163770816	-0.042970209	0.200353397	-1.280696331	-0.107067984	36	22	14

Supplementary Table S4. Full list of repurposable drugs

drug	initial rank	initial rank within top 30	GSEA (1 if ES<0, p-value<0.05)	ACE2 enzymatic activity	Spike-ACE2 protein-protein interaction (AlphaLISA)	SARS-CoV-2 cytopathic effect (NCATS)	SARS-CoV-2 cytopathic effect (ReFRAME)	positive efficacy in any in-vitro experiments	EHR	under_trials	prefer
Azithromycin	20	1	1	-1	-1	1	0	1	1	1	0.9998382324
Hydroxychloroquine	23	1	0	0	0	0	0	0	1	1	0.9987525694
Atorvastatin	44	0	1	0	0	0	0	0	1	1	0.9978606859
Acetaminophen	75	0	0	1	-1	-1	0	1	1	1	0.9977736695
Aspirin	189	0	-1	-1	-1	-1	0	0	1	1	0.989949518
Albuterol	270	0	0	0	0	0	0	0	1	0	0.9802454229
Melatonin	13	1	1	-1	-1	-1	0	0	-1	1	0.9786027904
Sirolimus	21	1	1	-1	-1	-1	0	0	0	1	0.9786027904
Nifedipine	9	1	1	-1	-1	-1	0	0	-1	1	0.9786027904
Ribavirin	22	1	0	-1	1	-1	0	1	0	1	0.9777499263
Chloroquine	15	1	0	-1	-1	1	1	1	0	1	0.9777499263
Lopinavir	14	1	0	-1	-1	1	0	1	0	1	0.9777499263
Teicoplanin	0	1	0	1	-1	1	0	1	0	1	0.9777499263
Remdesivir	7	1	0	-1	-1	1	1	1	-1	1	0.9777499263
Ivermectin	11	1	0	-1	-1	1	0	1	0	1	0.9777499263
Amlodipine	248	0	1	-1	-1	1	0	1	-1	1	0.9624068663
Celecoxib	55	0	1	1	-1	1	0	1	0	1	0.9624068663
Isotretinoin	60	0	1	1	-1	-1	0	1	0	1	0.9624068663
Chlorpromazine	182	0	1	-1	-1	1	0	1	0	1	0.9624068663
Itraconazole	34	0	1	-1	-1	1	0	1	0	1	0.9624068663
Progesterone	32	0	1	-1	-1	1	0	1	0	1	0.9624068663
Tenofovir	12	1	0	-1	-1	-1	0	0	0	1	0.9416406102
Mefloquine	1	1	0	0	0	0	0	0	0	1	0.9416406102
Ritonavir	4	1	0	-1	-1	-1	0	0	0	1	0.9416406102
Heparin	30	1	0	0	0	0	0	0	-1	1	0.9416406102
Cyclosporine	29	1	0	0	0	0	0	0	0	1	0.9416406102
Etoposide	25	1	0	-1	-1	-1	0	0	0	1	0.9416406102

Losartan	19	1	0	-1	-1	-1	0	0	-1	1	0.9416406102
Toremifene	2	1	1	0	0	0	0	0	0	0	0.9323913391
Cepharanthine	3	1	0	-1	-1	1	1	1	0	0	0.9298286253
Arbidol	5	1	0	-1	-1	1	0	1	0	0	0.9298286253
Valsartan	43	0	1	-1	-1	-1	0	0	-1	1	0.9038462396
Yohimbine	100	0	1	-1	-1	-1	0	0	0	1	0.9038462396
Clopidogrel	246	0	1	0	0	0	0	0	-1	1	0.9038462396
Naltrexone	136	0	1	0	0	0	0	0	0	1	0.9038462396
Amoxicillin	38	0	1	0	0	0	0	0	0	1	0.9038462396
Simvastatin	95	0	1	-1	-1	-1	0	0	-1	1	0.9038462396
Leflunomide	156	0	1	-1	-1	-1	0	0	0	1	0.9038462396
Thalidomide	175	0	1	-1	-1	-1	0	0	0	1	0.9038462396
Fluoxetine	103	0	1	0	0	0	0	0	-1	1	0.9038462396
Dexamethasone	80	0	1	-1	-1	-1	0	0	-1	1	0.9038462396
Lenalidomide	267	0	1	-1	-1	-1	0	0	0	1	0.9038462396
Eicosapentaenoic Acid	125	0	0	1	-1	-1	0	1	0	1	0.9003171987
Melphalan	127	0	0	1	-1	-1	0	1	0	1	0.9003171987
Famotidine	173	0	0	1	-1	-1	0	1	-1	1	0.9003171987
Omeprazole	91	0	0	1	-1	-1	0	1	-1	1	0.9003171987
Trimethoprim	112	0	0	1	-1	-1	0	1	0	1	0.9003171987
Dexmedetomidine	285	0	0	1	-1	-1	0	1	-1	1	0.9003171987
Clozapine	62	0	1	1	-1	1	0	1	0	0	0.8853163017
Tamibarotene	114	0	1	-1	-1	-1	1	1	0	0	0.8853163017
Tamoxifen	81	0	1	-1	-1	1	0	1	0	0	0.8853163017
Clonidine	275	0	1	1	-1	-1	0	1	-1	0	0.8853163017
Troglitazone	83	0	1	1	-1	1	0	1	0	0	0.8853163017
Ranitidine	162	0	1	1	-1	-1	0	1	0	0	0.8853163017
Fenofibrate	281	0	1	1	-1	-1	0	1	-1	0	0.8853163017
Tretinoin	56	0	1	0	0	0	1	1	0	0	0.8853163017
Amphetamine	28	1	0	0	0	0	0	0	0	0	0.8295094499
Gentamicins	27	1	0	0	0	0	0	0	0	0	0.8295094499
Doxorubicin	26	1	0	-1	-1	-1	0	0	0	0	0.8295094499

Valproic Acid	24	1	0	0	0	0	0	0	-1	0	0.8295094499
Betulinic Acid	18	1	0	-1	-1	-1	0	0	0	0	0.8295094499
Dactinomycin	17	1	0	0	0	0	0	0	0	0	0.8295094499
Antiviral Agents	16	1	0	0	0	0	0	0	0	0	0.8295094499
Emodin	10	1	0	0	0	0	0	0	0	0	0.8295094499
Immucillin A	8	1	0	0	0	0	0	0	0	0	0.8295094499
(2-Tert-Butoxy-1-(2-Cyclohexyl-1-(1-Formyl-2-(2-Oxopyrrolidin-3-Yl)Ethylcarbamoyl)Ethylcarbamoyl)Propyl)Carbamic Acid Benzyl Ester	6	1	0	0	0	0	0	0	0	0	0.8295094499
Tacrolimus	36	0	0	0	0	0	0	0	-1	1	0.7683199253
Quercetin	92	0	0	-1	-1	-1	0	0	0	1	0.7683199253
Hydrocortisone	98	0	0	-1	-1	-1	0	0	-1	1	0.7683199253
Methotrexate	84	0	0	-1	-1	-1	0	0	0	1	0.7683199253
Naproxen	120	0	0	0	0	0	0	0	0	1	0.7683199253
Folic Acid	82	0	0	-1	-1	-1	0	0	-1	1	0.7683199253
Nitric Oxide	115	0	0	0	0	0	0	0	0	1	0.7683199253
Candesartan	48	0	0	-1	-1	-1	0	0	0	1	0.7683199253
Iloprost	274	0	0	-1	-1	-1	0	0	0	1	0.7683199253
Spironolactone	53	0	0	-1	-1	-1	0	0	-1	1	0.7683199253
Doxycycline	222	0	0	-1	-1	-1	0	0	-1	1	0.7683199253
Isoflurane	219	0	0	-1	-1	-1	0	0	0	1	0.7683199253
Oxygen	45	0	0	0	0	0	0	0	0	1	0.7683199253
Vitamin A	264	0	0	0	0	0	0	0	0	1	0.7683199253
Metformin	263	0	0	-1	-1	-1	0	0	-1	1	0.7683199253
Bicalutamide	201	0	0	-1	-1	-1	0	0	0	1	0.7683199253
Oseltamivir	203	0	0	0	0	0	0	0	-1	1	0.7683199253

Prednisolone	210	0	0	-1	-1	-1	0	0	0	1	0.7683199253
Estradiol	57	0	0	-1	-1	-1	0	0	0	1	0.7683199253
Propofol	214	0	0	-1	-1	-1	0	0	-1	1	0.7683199253
Amiodarone	218	0	0	0	0	0	0	0	-1	1	0.7683199253
Ibuprofen	191	0	0	0	0	0	0	0	-1	1	0.7683199253
Alitretinoin	130	0	1	0	0	0	0	0	0	0	0.7392090996
Temozolomide	240	0	1	-1	-1	-1	0	0	0	0	0.7392090996
Bosentan	277	0	1	-1	-1	-1	0	0	0	0	0.7392090996
Bezafibrate	245	0	1	-1	-1	-1	0	0	0	0	0.7392090996
Riluzole	236	0	1	-1	-1	-1	0	0	0	0	0.7392090996
Sumatriptan	110	0	1	-1	-1	-1	0	0	0	0	0.7392090996
Levofloxacin	283	0	1	-1	-1	-1	0	0	-1	0	0.7392090996
Naringenin	284	0	1	0	0	0	0	0	0	0	0.7392090996
Propranolol	137	0	1	0	0	0	0	0	-1	0	0.7392090996
Triprolidine	149	0	1	-1	-1	-1	0	0	0	0	0.7392090996
Gemfibrozil	225	0	1	-1	-1	-1	0	0	0	0	0.7392090996
Theophylline	154	0	1	0	0	0	0	0	0	0	0.7392090996
Montelukast	170	0	1	0	0	0	0	0	-1	0	0.7392090996
Propylthiouracil	172	0	1	-1	-1	-1	0	0	0	0	0.7392090996
4-Methyl-N1-(3-Phenylpropyl)Benzene-1,2-Diamine	205	0	1	0	0	0	0	0	0	0	0.7392090996
Mercaptopurine	194	0	1	-1	-1	-1	0	0	0	0	0.7392090996
Glyburide	299	0	1	0	0	0	0	0	0	0	0.7392090996
Carbamazepine	297	0	1	-1	-1	-1	0	0	0	0	0.7392090996
Medroxyprogesterone Acetate	178	0	1	-1	-1	-1	0	0	0	0	0.7392090996
Epirubicin	150	0	1	0	0	0	0	0	0	0	0.7392090996
Flutamide	89	0	1	-1	-1	-1	0	0	0	0	0.7392090996
Irbesartan	79	0	1	-1	-1	-1	0	0	0	0	0.7392090996
Ampicillin	54	0	1	0	0	0	0	0	0	0	0.7392090996
Betamethasone	69	0	1	0	0	0	0	0	0	0	0.7392090996

Mycophenolic Acid	153	0	0	-1	1	1	0	1	0	0	0.7314329993
Raloxifene Hydrochloride	94	0	0	-1	-1	1	0	1	0	0	0.7314329993
Dacarbazine	250	0	0	1	-1	-1	0	1	0	0	0.7314329993
Fluorouracil	51	0	0	-1	1	-1	0	1	0	0	0.7314329993
Promethazine	273	0	0	-1	-1	1	0	1	-1	0	0.7314329993
Efavirenz	143	0	0	-1	-1	1	0	1	0	0	0.7314329993
Mifepristone	58	0	0	-1	-1	1	0	1	0	0	0.7314329993
Pomalidomide	234	0	0	-1	1	-1	0	1	0	0	0.7314329993
Rosiglitazone	122	0	0	-1	-1	1	0	1	0	0	0.7314329993
Olmesartan Medoxomil	42	0	0	1	-1	-1	0	1	0	0	0.7314329993
Acetazolamide	78	0	0	-1	1	-1	0	1	-1	0	0.7314329993
Zidovudine	164	0	0	1	-1	-1	0	1	0	0	0.7314329993
Cilostazol	294	0	0	-1	1	-1	0	1	0	0	0.7314329993
Mefenamic Acid	295	0	0	1	-1	-1	0	1	0	0	0.7314329993
Ospemifene	113	0	0	-1	-1	1	0	1	0	0	0.7314329993
Gemcitabine	146	0	0	1	-1	-1	0	1	0	0	0.7314329993
Terbinafine	111	0	0	1	-1	-1	0	1	0	0	0.7314329993
Benazepril	31	0	0	-1	-1	1	0	1	0	0	0.7314329993
Dipyridamole	258	0	-1	-1	-1	-1	0	0	0	1	0.6649908863
Indomethacin	141	0	-1	-1	-1	-1	0	0	0	1	0.6649908863
Telmisartan	65	0	-1	-1	-1	-1	0	0	0	1	0.6649908863
Disulfiram	90	0	-1	-1	-1	1	0	1	0	0	0.6197935439
Cyclophosphamide	73	0	-1	1	-1	-1	0	1	0	0	0.6197935439
Triclosan	61	0	-1	1	-1	-1	0	1	0	0	0.6197935439
Ketoconazole	133	0	-1	1	-1	-1	0	1	0	0	0.6197935439
Diethylstilbestrol	40	0	-1	-1	-1	1	0	1	0	0	0.6197935439
Albendazole	123	0	-1	1	-1	-1	0	1	0	0	0.6197935439
Mannitol	241	0	0	0	0	0	0	0	0	0	0.5
Chloroquine Diphosphate	59	0	0	0	0	0	0	0	0	0	0.5

Isoflurophate	242	0	0	0	0	0	0	0	0	0	0.5
Acetamide	238	0	0	0	0	0	0	0	0	0	0.5
Digoxin	239	0	0	-1	-1	-1	0	0	-1	0	0.5
Allergens	244	0	0	0	0	0	0	0	0	0	0.5
Azathioprine	247	0	0	-1	-1	-1	0	0	0	0	0.5
Pioglitazone	52	0	0	0	0	0	0	0	0	0	0.5
Amphotericin B	243	0	0	-1	-1	-1	0	0	0	0	0.5
Colforsin	204	0	0	0	0	0	0	0	0	0	0.5
Aspartame	237	0	0	0	0	0	0	0	0	0	0.5
Neomycin	235	0	0	0	0	0	0	0	0	0	0.5
Nitroglycerin	213	0	0	0	0	0	0	0	-1	0	0.5
Sunitinib	67	0	0	0	0	0	0	0	0	0	0.5
Active Hexose Correlated Compound	216	0	0	0	0	0	0	0	0	0	0.5
Amlexanox	217	0	0	-1	-1	-1	0	0	0	0	0.5
Atrazine	66	0	0	0	0	0	0	0	0	0	0.5
Olmesartan	64	0	0	-1	-1	-1	0	0	0	0	0.5
Penicillamine	223	0	0	0	0	0	0	0	0	0	0.5
Ammonia	224	0	0	0	0	0	0	0	0	0	0.5
Bisphenol A	63	0	0	0	0	0	0	0	0	0	0.5
Codeine	226	0	0	0	0	0	0	0	0	0	0.5
Epinephrine	227	0	0	-1	-1	-1	0	0	-1	0	0.5
Zoledronic Acid	228	0	0	0	0	0	0	0	0	0	0.5
Fluticasone	229	0	0	0	0	0	0	0	0	0	0.5
Phenformin	230	0	0	0	0	0	0	0	0	0	0.5
Water	231	0	0	0	0	0	0	0	0	0	0.5
Cobalt	232	0	0	0	0	0	0	0	0	0	0.5
Zileuton	233	0	0	-1	-1	-1	0	0	0	0	0.5
Norepinephrine	249	0	0	0	0	0	0	0	-1	0	0.5
Moxifloxacin	260	0	0	0	0	0	0	0	0	0	0.5
Carnitine	251	0	0	0	0	0	0	0	0	0	0.5
Rifaximin	252	0	0	-1	-1	-1	0	0	0	0	0.5
Silver	41	0	0	0	0	0	0	0	0	0	0.5
Sodium Chloride	278	0	0	0	0	0	0	0	0	0	0.5
Vitamin K 3	280	0	0	0	0	0	0	0	0	0	0.5

Ofloxacin	282	0	0	0	0	0	0	0	0	0	0.5
Furan	39	0	0	0	0	0	0	0	0	0	0.5
Gadodiamide	37	0	0	0	0	0	0	0	0	0	0.5
Bleomycin	35	0	0	0	0	0	0	0	0	0	0.5
Alpha-Tocopherol	286	0	0	0	0	0	0	0	0	0	0.5
Fluvastatin	287	0	0	0	0	0	0	0	0	0	0.5
Niacinamide	288	0	0	0	0	0	0	0	0	0	0.5
Formoterol Fumarate	290	0	0	0	0	0	0	0	0	0	0.5
Ursodeoxycholic Acid	291	0	0	0	0	0	0	0	0	0	0.5
Mechlorethamine	292	0	0	0	0	0	0	0	0	0	0.5
Pamidronate	293	0	0	0	0	0	0	0	0	0	0.5
Penicillin V	296	0	0	0	0	0	0	0	0	0	0.5
Ethinyl Estradiol	33	0	0	0	0	0	0	0	0	0	0.5
Methimazole	298	0	0	-1	-1	-1	0	0	0	0	0.5
Gliclazide	276	0	0	-1	-1	-1	0	0	0	0	0.5
Ascorbic Acid	46	0	0	0	0	0	0	0	-1	0	0.5
Vardenafil Dihydrochloride	272	0	0	0	0	0	0	0	0	0	0.5
Vinblastine	261	0	0	0	0	0	0	0	0	0	0.5
Vitamin E	253	0	0	0	0	0	0	0	0	0	0.5
Hesperetin	254	0	0	0	0	0	0	0	0	0	0.5
Zomepirac	255	0	0	0	0	0	0	0	0	0	0.5
Tigecycline	256	0	0	-1	-1	-1	0	0	0	0	0.5
Sesamol	257	0	0	0	0	0	0	0	0	0	0.5
Alendronate	259	0	0	0	0	0	0	0	0	0	0.5
Floxacillin	211	0	0	0	0	0	0	0	0	0	0.5
Apigenin	262	0	0	0	0	0	0	0	0	0	0.5
Phenobarbital	271	0	0	0	0	0	0	0	-1	0	0.5
Ethanol	50	0	0	0	0	0	0	0	0	0	0.5
Taurocholic Acid	265	0	0	0	0	0	0	0	0	0	0.5
Sulfapyridine	266	0	0	-1	-1	-1	0	0	0	0	0.5
Dapsone	49	0	0	-1	-1	-1	0	0	0	0	0.5
Salmeterol Xinafoate	268	0	0	-1	-1	-1	0	0	0	0	0.5

Resveratrol	269	0	0	-1	-1	-1	0	0	0	0	0.5
Arsenic Trioxide	47	0	0	0	0	0	0	0	0	0	0.5
Carmustine	212	0	0	-1	-1	-1	0	0	0	0	0.5
Cimetidine	300	0	0	-1	-1	-1	0	0	0	0	0.5
Cocaine	68	0	0	0	0	0	0	0	0	0	0.5
Ciprofloxacin	151	0	0	-1	-1	-1	0	0	-1	0	0.5
Fulvestrant	139	0	0	-1	-1	-1	0	0	0	0	0.5
Chlorine	140	0	0	0	0	0	0	0	0	0	0.5
Imatinib Mesylate	142	0	0	0	0	0	0	0	0	0	0.5
Rifampin	144	0	0	0	0	0	0	0	0	0	0.5
Menthol	145	0	0	0	0	0	0	0	0	0	0.5
Permethrin	147	0	0	-1	-1	-1	0	0	0	0	0.5
Phenytoin	148	0	0	0	0	0	0	0	0	0	0.5
Perindopril	93	0	0	-1	-1	-1	0	0	0	0	0.5
Trovafloxacin	152	0	0	0	0	0	0	0	0	0	0.5
Succimer	96	0	0	0	0	0	0	0	0	0	0.5
Topotecan	155	0	0	0	0	0	0	0	0	0	0.5
Beclomethasone	88	0	0	-1	-1	-1	0	0	0	0	0.5
Lansoprazole	157	0	0	0	0	0	0	0	-1	0	0.5
Estrogens	158	0	0	0	0	0	0	0	0	0	0.5
Dextroamphetamine	159	0	0	0	0	0	0	0	0	0	0.5
Arsenite	160	0	0	0	0	0	0	0	0	0	0.5
Dronabinol	161	0	0	0	0	0	0	0	0	0	0.5
Pentamidine	87	0	0	0	0	0	0	0	0	0	0.5
Mitomycin	138	0	0	0	0	0	0	0	0	0	0.5
Tetrachlorodibenzodioxin	135	0	0	0	0	0	0	0	0	0	0.5
Dextromethorphan	165	0	0	0	0	0	0	0	-1	0	0.5
Dust	121	0	0	0	0	0	0	0	0	0	0.5
Ethambutol	106	0	0	0	0	0	0	0	0	0	0.5
Levonorgestrel	105	0	0	0	0	0	0	0	0	0	0.5
Cerivastatin	104	0	0	0	0	0	0	0	0	0	0.5
Cisplatin	116	0	0	0	0	0	0	0	0	0	0.5
Nelfinavir	117	0	0	0	0	0	0	0	0	0	0.5
Diclofenac	118	0	0	0	0	0	0	0	0	0	0.5
Methadone	119	0	0	0	0	0	0	0	0	0	0.5

Pyridostigmine Bromide	102	0	0	-1	-1	-1	0	0	0	0	0.5
Meloxicam	124	0	0	0	0	0	0	0	0	0	0.5
Rofecoxib	134	0	0	-1	-1	-1	0	0	0	0	0.5
Metoprolol	101	0	0	0	0	0	0	0	-1	0	0.5
Pravastatin	126	0	0	0	0	0	0	0	-1	0	0.5
Vancomycin	99	0	0	0	0	0	0	0	-1	0	0.5
Vincristine	128	0	0	0	0	0	0	0	0	0	0.5
Nicotine	129	0	0	0	0	0	0	0	-1	0	0.5
Methoxychlor	97	0	0	0	0	0	0	0	0	0	0.5
Nitrogen	131	0	0	0	0	0	0	0	0	0	0.5
Dihydrotestosterone	132	0	0	0	0	0	0	0	0	0	0.5
Diphenhydramine	209	0	0	0	0	0	0	0	-1	0	0.5
Valdecoxib	163	0	0	-1	-1	-1	0	0	0	0	0.5
Daunorubicin	166	0	0	-1	-1	-1	0	0	0	0	0.5
Allopurinol	188	0	0	-1	-1	-1	0	0	0	0	0.5
Bisoprolol	190	0	0	0	0	0	0	0	0	0	0.5
Irinotecan	167	0	0	0	0	0	0	0	0	0	0.5
Enalaprilat	193	0	0	0	0	0	0	0	0	0	0.5
Polycyclic Aromatic Hydrocarbons	72	0	0	0	0	0	0	0	0	0	0.5
Tacrine	195	0	0	0	0	0	0	0	0	0	0.5
Fexofenadine	196	0	0	0	0	0	0	0	0	0	0.5
Sodium	197	0	0	0	0	0	0	0	0	0	0.5
Erythromycin	198	0	0	0	0	0	0	0	0	0	0.5
Nsc 23766	199	0	0	0	0	0	0	0	0	0	0.5
Roxithromycin	200	0	0	-1	-1	-1	0	0	0	0	0.5
Streptozocin	71	0	0	0	0	0	0	0	0	0	0.5
Dydrogesterone	202	0	0	-1	-1	-1	0	0	0	0	0.5
Oxaliplatin	70	0	0	-1	-1	-1	0	0	0	0	0.5
Quinapril	109	0	0	0	0	0	0	0	0	0	0.5
Kojic Acid	206	0	0	-1	-1	-1	0	0	0	0	0.5
Amiloride	207	0	0	0	0	0	0	0	0	0	0.5
Terfenadine	208	0	0	-1	-1	-1	0	0	0	0	0.5
Silicon Dioxide	74	0	0	0	0	0	0	0	0	0	0.5
Tetracycline	192	0	0	0	0	0	0	0	0	0	0.5

Isoniazid	187	0	0	-1	-1	-1	0	0	0	0	0.5
Dasatinib	77	0	0	-1	-1	-1	0	0	0	0	0.5
Indinavir	168	0	0	0	0	0	0	0	0	0	0.5
Sulfasalazine	169	0	0	-1	-1	-1	0	0	0	0	0.5
Testosterone	86	0	0	0	0	0	0	0	0	0	0.5
Morphine	171	0	0	0	0	0	0	0	-1	0	0.5
Chenodeoxycholic Acid	85	0	0	0	0	0	0	0	0	0	0.5
Apomorphine	174	0	0	-1	-1	-1	0	0	0	0	0.5
Polymyxin B	186	0	0	0	0	0	0	0	0	0	0.5
Isoproterenol	177	0	0	0	0	0	0	0	0	0	0.5
Vinorelbine	176	0	0	0	0	0	0	0	0	0	0.5
Furosemide	179	0	0	-1	-1	-1	0	0	-1	0	0.5
Calcitriol	180	0	0	-1	-1	-1	0	0	-1	0	0.5
Amitriptyline	181	0	0	0	0	0	0	0	-1	0	0.5
Erlotinib Hydrochloride	76	0	0	0	0	0	0	0	0	0	0.5
Oxytetracycline	183	0	0	-1	-1	-1	0	0	0	0	0.5
Crack Cocaine	184	0	0	0	0	0	0	0	0	0	0.5
Thioctic Acid	185	0	0	-1	-1	-1	0	0	0	0	0.5
Warfarin	289	0	-1	0	0	0	0	0	-1	0	0.3744358979
Mesalamine	221	0	-1	0	0	0	0	0	0	0	0.3744358979
Lidocaine	220	0	-1	-1	-1	-1	0	0	-1	0	0.3744358979
Genistein	215	0	-1	-1	-1	-1	0	0	0	0	0.3744358979
Enalapril	279	0	-1	0	0	0	0	0	0	0	0.3744358979
Bortezomib	107	0	-1	-1	-1	-1	0	0	0	0	0.3744358979
Clofibrate	108	0	-1	-1	-1	-1	0	0	0	0	0.3744358979

Supplementary Table S5. Full list of drug combinations from the top drugs.

Drug A	Drug B	# COVID-19 genes that Drug A hits	# COVID-19 genes that Drug B hits	# COVID-19 genes that either Drug A or B hit	# COVID-19 genes that both Drug A and Drug B hit
ETOPOSIDE	SIROLIMUS	2	22	24	0
MEFLOQUINE	SIROLIMUS	1	22	23	0
LOSARTAN	RIBAVIRIN	12	6	18	0
ACETAMINOPHEN	CHLOROQUINE	3	11	14	0
ETOPOSIDE	LOSARTAN	2	12	14	0
HYDROXYCHLOROQUINE	MELATONIN	4	10	14	0
ACETAMINOPHEN	MELATONIN	3	10	13	0
ATORVASTATIN	CHLOROQUINE	2	11	13	0
CHLOROQUINE	LOPINAVIR	11	2	13	0
LOSARTAN	MEFLOQUINE	12	1	13	0
IVERMECTIN	RIBAVIRIN	6	6	12	0
ETOPOSIDE	MELATONIN	2	10	12	0
AZITHROMYCIN	MELATONIN	2	10	12	0
ATORVASTATIN	MELATONIN	2	10	12	0
CHLOROQUINE	HEPARIN	11	1	12	0
HEPARIN	MELATONIN	1	10	11	0
MEFLOQUINE	MELATONIN	1	10	11	0
ASPIRIN	RIBAVIRIN	4	6	10	0
HYDROXYCHLOROQUINE	RIBAVIRIN	4	6	10	0
HYDROXYCHLOROQUINE	IVERMECTIN	4	6	10	0
NIFEDIPINE	RIBAVIRIN	4	6	10	0
ACETAMINOPHEN	RIBAVIRIN	3	6	9	0
HYDROXYCHLOROQUINE	NIFEDIPINE	4	4	8	0
LOPINAVIR	RIBAVIRIN	2	6	8	0
HYDROXYCHLOROQUINE	TENOFOVIR	4	4	8	0
ETOPOSIDE	RIBAVIRIN	2	6	8	0
IVERMECTIN	LOPINAVIR	6	2	8	0
AZITHROMYCIN	RIBAVIRIN	2	6	8	0
ATORVASTATIN	IVERMECTIN	2	6	8	0
ATORVASTATIN	RIBAVIRIN	2	6	8	0
ASPIRIN	HYDROXYCHLOROQUINE	4	4	8	0
CYCLOSPORINE	RIBAVIRIN	2	6	8	0
MEFLOQUINE	RIBAVIRIN	1	6	7	0
HEPARIN	IVERMECTIN	1	6	7	0

HEPARIN	RIBAVIRIN	1	6	7	0
ACETAMINOPHEN	TENOFOVIR	3	4	7	0
ACETAMINOPHEN	HYDROXYCHLOROQUINE	3	4	7	0
ACETAMINOPHEN	NIFEDIPINE	3	4	7	0
CYCLOSPORINE	TENOFOVIR	2	4	6	0
LOPINAVIR	TENOFOVIR	2	4	6	0
HYDROXYCHLOROQUINE	LOPINAVIR	4	2	6	0
ETOPOSIDE	HYDROXYCHLOROQUINE	2	4	6	0
ETOPOSIDE	NIFEDIPINE	2	4	6	0
CYCLOSPORINE	HYDROXYCHLOROQUINE	2	4	6	0
AZITHROMYCIN	HYDROXYCHLOROQUINE	2	4	6	0
ETOPOSIDE	TENOFOVIR	2	4	6	0
ATORVASTATIN	NIFEDIPINE	2	4	6	0
ATORVASTATIN	HYDROXYCHLOROQUINE	2	4	6	0
ATORVASTATIN	TENOFOVIR	2	4	6	0
ASPIRIN	ETOPOSIDE	4	2	6	0
AZITHROMYCIN	TENOFOVIR	2	4	6	0
ASPIRIN	CYCLOSPORINE	4	2	6	0
ASPIRIN	AZITHROMYCIN	4	2	6	0
ASPIRIN	ATORVASTATIN	4	2	6	0
HEPARIN	NIFEDIPINE	1	4	5	0
MEFLOQUINE	TENOFOVIR	1	4	5	0
MEFLOQUINE	NIFEDIPINE	1	4	5	0
HYDROXYCHLOROQUINE	MEFLOQUINE	4	1	5	0
HEPARIN	TENOFOVIR	1	4	5	0
ASPIRIN	HEPARIN	4	1	5	0
ACETAMINOPHEN	ATORVASTATIN	3	2	5	0
ACETAMINOPHEN	ETOPOSIDE	3	2	5	0
ACETAMINOPHEN	CYCLOSPORINE	3	2	5	0
ACETAMINOPHEN	AZITHROMYCIN	3	2	5	0
ACETAMINOPHEN	LOPINAVIR	3	2	5	0
ASPIRIN	MEFLOQUINE	4	1	5	0
ETOPOSIDE	LOPINAVIR	2	2	4	0
CYCLOSPORINE	LOPINAVIR	2	2	4	0
AZITHROMYCIN	LOPINAVIR	2	2	4	0
ATORVASTATIN	ETOPOSIDE	2	2	4	0
ATORVASTATIN	AZITHROMYCIN	2	2	4	0
ACETAMINOPHEN	HEPARIN	3	1	4	0
ACETAMINOPHEN	MEFLOQUINE	3	1	4	0

ATORVASTATIN	CYCLOSPORINE	2	2	4	0
ATORVASTATIN	MEFLOQUINE	2	1	3	0
ATORVASTATIN	HEPARIN	2	1	3	0
AZITHROMYCIN	HEPARIN	2	1	3	0
CYCLOSPORINE	HEPARIN	2	1	3	0
ETOPOSIDE	HEPARIN	2	1	3	0
HEPARIN	LOPINAVIR	1	2	3	0
LOPINAVIR	MEFLOQUINE	2	1	3	0
HEPARIN	MEFLOQUINE	1	1	2	0
